# Up‐regulation of TβRIII facilitates the osteogenesis of supraspinous ligament‐derived fibroblasts from patients with ankylosing spondylitis

**DOI:** 10.1111/jcmm.16262

**Published:** 2021-01-06

**Authors:** Ying Zhang, Wu‐gui Chen, Si‐zhen Yang, Hao Qiu, Xu Hu, Yi‐yun Qiu, Xuan Wen, Yue Zhou, Tong‐wei Chu

**Affiliations:** ^1^ Department of Orthopaedics Xinqiao Hospital Army Military Medical University Chongqing China

**Keywords:** ankylosing spondylitis, bone morphogenetic protein 2, osteogenesis, supraspinous ligament fibroblasts, transforming growth factor β1, type III transforming growth factor β receptor

## Abstract

Spinal supraspinous ligament (SL) osteogenesis is the key risk of ankylosing spondylitis (AS), with an unclear pathogenesis. We previously found that transforming growth factor β1 (TGF‐β1), bone morphogenetic proteins (eg BMP2) and type III TGF‐β1 receptor (TβRIII) expression were markedly up‐regulated in AS‐SLs. However, the roles of these closely related molecules in AS are unknown. Here, we showed that BMP2, TGF‐β1, TβRIII and S100A4 (a fibroblast marker) were abundant in active osteogenic AS‐SL tissues. In vitro, AS‐SL fibroblasts (AS‐SLFs) showed high BMP2, TGF‐β1 and TβRIII expression and auto‐osteogenic capacity. We further evaluated the role of TβRIII in the osteogenesis of normal SLFs. BMP2 combined with TGF‐β1 induced the osteogenesis of TβRIII‐overexpressing SLFs, but the activity was lost in SLFs upon TβRIII knockdown. Moreover, our data suggested that BMP2 combined with TGF‐β1 significantly activated both TGF‐β1/Smad signalling and BMP2/Smad/RUNX2 signalling to induce osteogenesis of SLFs with TβRIII up‐regulation. Furthermore, our multi‐strategy molecular interaction analysis approach indicated that TGF‐β1 presented BMP2 to TβRIII, sequentially facilitating BMP2 recognition by BMPR1A and promoting the osteogenesis of TβRIII‐overexpressing SLFs. Collectively, our results indicate that TGF‐β1 combined with BMP2 may participate in the osteogenic differentiation of AS‐SLF by acting on up‐regulated TβRIII, resulting in excessive activation of both TGF‐β1/Smad and BMP2/BMPR1A/Smad/RUNX2 signalling.

## 1 BACKGROUND

Ankylosing spondylitis (AS) is the prototypic phenotype of a class of immune‐related spondyloarthropathies that affect the spine, sacroiliac joint and hip joint. AS commonly causes spinal rigidity, which manifests as inflammatory pain, spinal fusion, structural and functional impairment of the sacroiliac joint and hip joint. Moreover, the complications of AS include reactive arthritis, Crohn's disease, ulcerative colitis, psoriatic arthritis and acute anterior uveitis. The late stage of AS may lead to spinal fusion and the development of a ‘bamboo‐like’ spine, ultimately resulting in a high risk of disability. Therefore, investigating the pathogenesis of and therapeutic strategies for AS is valuable.

The pathogenesis of AS, which is complex and incompletely understood, involves changes in spinal joints, cartilage, tendons and ligaments. Heterotopic osteogenesis of supraspinous ligaments (SLs) is the key risk of AS, although the pathogenesis remains unclear. A previous study showed that transforming growth factor β1 (TGF‐β1) and bone morphogenetic proteins (eg BMP2) are up‐regulated in peripheral blood from AS patients.^1^ Our previous reports further indicated that the expression of TGF‐β1, BMP2 and the type III TGF‐β receptor (TβRIII) was significantly increased in AS‐SLs compared with SLs from control patients.^2^, ^3^ However, whether TGF‐β1, BMP2 and TβRIII participate in the osteogenesis of AS‐SLs is unclear.

Transforming growth factor β1 and BMP2 are members of the TGF‐β superfamily. Members of this family form a complex with the TGF‐β receptor family, including TβRI, TβII and TβRIII, thereby regulating upstream Smad signalling.^4^ Among them, TβRIII (also known as β‐proteoglycan), an abundant membrane‐anchoring protein, was originally thought to be an auxiliary receptor of the TGF‐β superfamily. Previous reports indicate that TβRIII plays a critical role in TGF‐β/Smad signalling.^5^ A report from another group suggests that BMP2, BMP4 and BMP7 can bind to TβRIII using a biosensor method and that loss of TβRIII markedly decreases BMP2 activity.^6^, ^7^ Moreover, a recent report suggests that TGF‐β1 and BMP2 co‐regulate TβRIII to activate epithelial‐mesenchymal transition (EMT) and invasion in epicardial cells, further suggesting that TGF‐β1 and BMP2 can simultaneously act on TβRIII.^7^ However, whether TGF‐β1 and BMP2 co‐regulate TβRIII in AS‐SLs is unknown.

Unexpectedly, we found that some fibroblast‐like cells isolated from AS‐SLs showed auto‐osteogenic capacity. These cells expressed high levels of fibroblast‐specific protein 1 (Fsp1, also known as S100A4), which is a specific marker of fibroblasts.^8^ Therefore, we hypothesized that TGF‐β1 and BMP2 may participate in the osteogenesis of SL fibroblasts (SLFs) from patients with AS (AS‐SLFs) through cooperatively acting on TβRIII. This study aimed to further unveil the role of TβRIII in the osteogenesis of AS‐SLFs and provide a new target for developing promising therapies.

## 2 EXPERIMENTAL SECTION

### 2.1 Sampling

Ankylosing spondylitis‐supraspinous ligaments were obtained via spinal orthopaedic surgery from 12 patients (nine males and three females; mean age, 30.2 years) who were in the active stage according to the American Rheumatism Association 1987 revised criteria.^9^ Control supraspinous ligaments (C‐SLs) were obtained via reattachment surgery from four patients (two males and two females; mean age, 31.5 years) with spinal fractures. SLs were washed with phosphate‑buffered saline (PBS; 0.01 mmol/L; pH 7.2) and were then either stored in liquid nitrogen or fixed with 4% (*m/v*) paraformaldehyde solution and embedded in paraffin. All specimens were obtained with the approval of the ethics committee of Xinqiao Hospital and patient consent.

### 2.2 Histological observations

Haematoxylin and eosin (HE) staining was applied to observe the pathologic features of SLs from AS or control patients as described in our previous report.^3^ To detect protein expression in SLs, immunohistochemistry (IHC) was carried out with antibodies against TGF‐β1, BMP2 (Abcam), S100A4, TβRIII, phospho‐Smad2^Ser465/467^/3^Ser423/425^ (p‐Smad2/3), Smad4, phospho‐Smad1^Ser206^ (p‐Smad1) and runt‐related transcription factor 2 (RUNX2; Cell Signaling) as reported previously.^3^ The histological images were acquired under a light microscope and analysed by ImageJ.

### 2.3 Cell cultures

The tissue piece method was applied to isolate supraspinous ligament fibroblasts (SLFs) from AS patients (AS‐SLFs) or spinal fracture patients (C‐SLFs) according to a previously reported protocol.^10^ Briefly, ligaments were washed with Hank's Balanced Salt Solution (HBSS; pH 7.4), minced and cultured in Dulbecco's modified Eagle's medium (DMEM; Gibco) containing 10% (*v/v*) foetal bovine serum (FBS; Gibco), penicillin (100 U/mL) and streptomycin (100 µg/mL) for 14 days. After SLFs grew, the tissue pieces were removed, and the cells were cultured in complete DMEM.

### 2.4 Overexpression and RNA interference

3′‐Flag tagged *TβRIII* (GenBank No. NM_001195684) was synthesized and sub‐cloned into the vector pcDNA3.1 (Thermo Fisher). The recombinant plasmid used for overexpression was verified by DNA sequencing. Gene knockdown was performed using small interfering RNA (siRNA; Thermo Fisher). The plasmid was transformed into C‐SLFs to obtain C‐SLFs with *TβRIII* overexpression using Lipofectamine 3000 (Thermo Fisher) according to the user manuals. Similarly, *TβRIII* siRNA was introduced into C‐SLFs with *TβRIII* overexpression to obtain C‐SLFs with *TβRIII* knockdown.

### 2.5 Osteogenic analysis

Supraspinous ligament fibroblasts cultured in 24‐well plates (1 × 10^4^ cells/well) were treated as indicated and differentiated in DMEM containing FBS (10%; *m/v*), β‐sodium glycerophosphate (10 mmol/L), dexamethasone (0.1 μmol/L) and vitamin C (30 mmol/L) for 14 days. Then, the cells were further used for mineralization assays or alkaline phosphatase (ALP) activity assays. Mineralization was determined using Alizarin red S staining (Sigma) as described previously.^11^ In brief, cells were fixed in 95% ethanol, washed with water, incubated with Alizarin red S solution (40 mmol/L pH 4.2) for 15 minutes and washed again with water. The stained cells were imaged using a Leica light microscope (Leica). To detect ALP activity, cells were harvested with cold PBS and gently sonicated. Samples containing equal amounts of protein were used to detect ALP activity using an ALP detection kit (Beyotime).

### 2.6 Immunoblotting

Immunoblotting (IB) was performed to detect protein expression as described previously.^10^ Briefly, equal amounts of protein extracted from tissues or cells were separated by sodium dodecyl sulphate‐polyacrylamide gel electrophoresis (SDS‐PAGE). The protein bands were transferred to polyvinylidene fluoride (PVDF) membranes, blocked with 5% (*m/v*) skim milk and probed with the corresponding primary antibodies and horseradish peroxidase (HRP)‐conjugated secondary antibodies (Abcam). Chemiluminescence imaging was carried out with Clarity Western ECL substrate (Bio‐Rad) in a ChemiDoc™ Touch Imaging System (Bio‐Rad). ImageJ was used to calculate protein expression ratios normalized to the expression of GAPDH and that in the control group.

### 2.7 Immunoprecipitation

Immunoprecipitation (IP) was performed using a Protein A/G Magnetic Beads kit (Thermo Fisher) according to the user manual. C‐SLFs grown in dishes were transfected with the flag‐*TβRIII* construct using Lipofectamine 3000. After treatment with TGF‐β1 and/or BMP2 as indicated, the cell lysate was harvested using the cell lysis‐wash buffer supplied in the kit, and 1 mg of protein was incubated with primary antibodies or anti‐mouse IgG (negative control) overnight at 4°C. Immune complexes were captured by the Protein A/G beads and detected by IB.

### 2.8 Immunofluorescence

Supraspinous ligament fibroblasts grown on glass slides in a 24‐well plate (1 × 10^4^ cells/well) were treated as indicated, fixed with 4% (*m/v*) paraformaldehyde solution and blocked with PBS containing 1% (*m/v*) skim milk and 0.1% (*m/v*) Triton X‐100. The cell cytoskeleton was stained with rhodamine‐phalloidin (Sigma). TβRIII was probed with a rabbit anti‐TβRIII antibody (Abcam) followed by an Alexa Fluor 555‐conjugated secondary antibody (Sigma; red fluorescence). TGF‐β1 and BMP2 were probed with mouse primary antibodies (Abcam) followed by an Alexa Fluor 488‐conjugated secondary antibody (Sigma; green fluorescence). Cell nuclei were stained with DAPI. Fluorescence images were acquired with a laser confocal microscope (Zeiss). ImageJ was used to calculate the cell area, mean fluorescence intensity and the Pearson correlation coefficient (*Rr*) to indicate colocalization.

### 2.9 Statistics

Data in histograms are shown as the means ± SDs. Student's *t* test was used for pairwise comparisons. Differences with a *P* value of <.05 were considered statistically significant, <.01 and highly statistically significant.

## 3 RESULTS

### 3.1 S100A4, TβRIII, TGF‐β1 and BMP2 are up‐regulated in AS‐SLs

**FIGURE 1:**
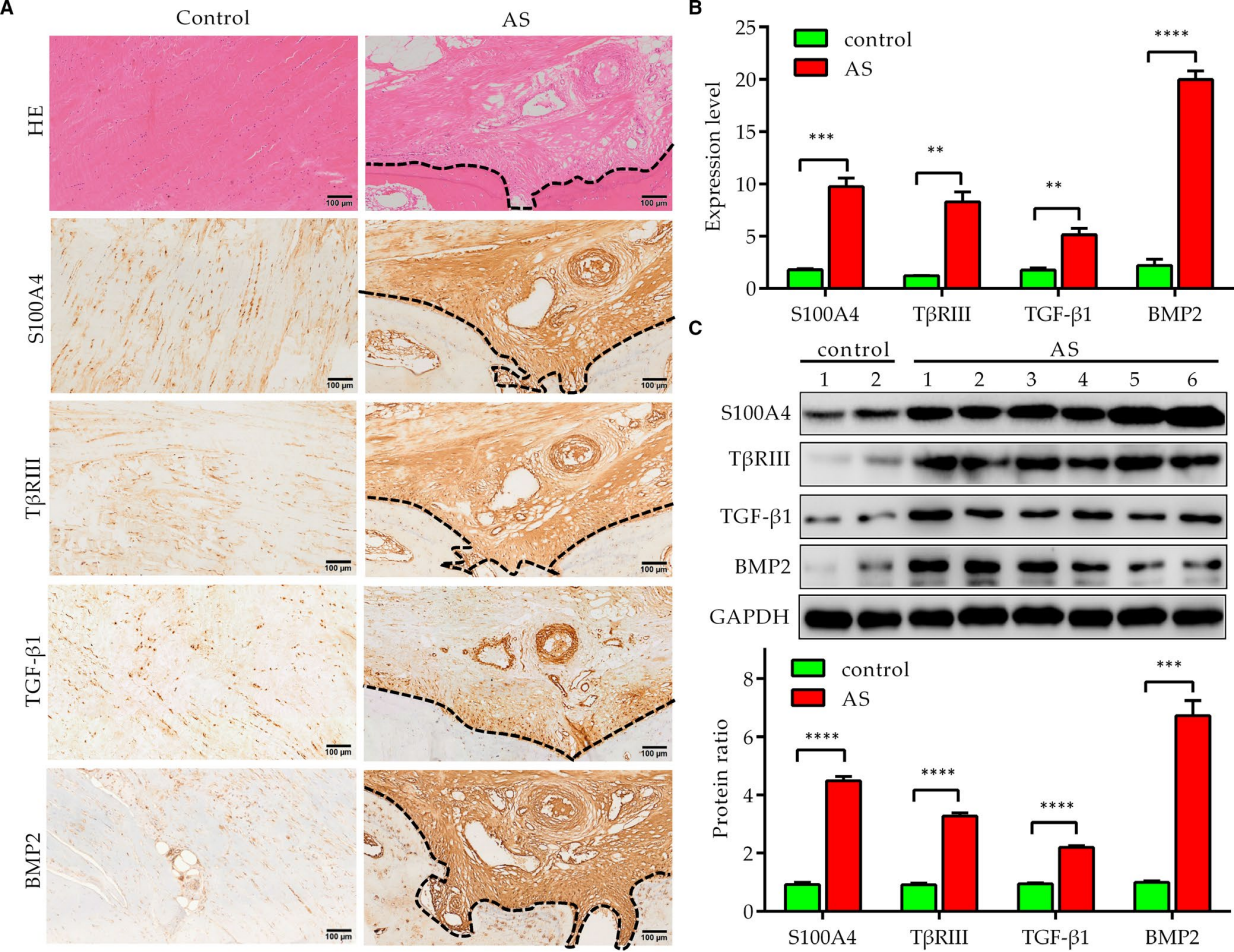
Supraspinous ligaments of AS patients exhibit up‐regulation of S100A4, TβRIII, TGF‐β1 and BMP2. A, Sections of supraspinous ligaments from patients with spinal fracture (control) or AS were stained with haematoxylin and eosin (HE) or subjected to immunohistochemistry (IHC) using antibodies against S100A4, TβRIII, TGF‐β1 and BMP2. The histological changes were evaluated under a light microscope (bar = 100 μm). The dotted lines indicate the edge of the ectopic bone. B, Expression levels as indicated by IHC. Expression levels were detected by measuring the positive staining area in ImageJ and were calculated by normalization to a control. C, Total tissue lysates of samples as described in (A) were examined by immunoblotting using the corresponding antibodies. The histograms indicate the protein expression ratios normalized to GAPDH expression. The dotted line indicates the edge of the ectopic bone. **P < .01, ***P < .001 and ****P < .0001 (control: n = 4; AS: n = 12)

The HE staining results showed that the fibres of AS‐SLs were broken and disordered, with collagen deposition, vascular proliferation and ectopic bone formation (as shown in dotted line), indicating an osteogenic morphology (Figure 1A). The immunohistochemical results suggested that S100A4 (a fibroblast marker), TβRIII, TGF‐β1 and BMP2 were up‐regulated in the tissue near the ectopic bone in the contiguous sections, and the expression region was very consistent (Figure 1A,B). To further assess the expression of these proteins, total protein was extracted from AS‐SLs and C‐SLs. As expected, the IB results also showed that the expression of S100A4, TβRIII, TGF‐β1 and BMP2 was markedly increased in AS‐SLs (Figure 1C). Together, these results suggest that TβRIII, TGF‐β1 and BMP2 are up‐regulated in AS‐SLs with abundant fibroblasts. However, further studies are needed to determine whether these proteins are expressed in the additional fibroblasts.

### 3.2 AS‐SLFs exhibit auto‐osteogenic capacity and up‐regulation of TβRIII, TGF‐β1 and BMP2

**FIGURE 2:**
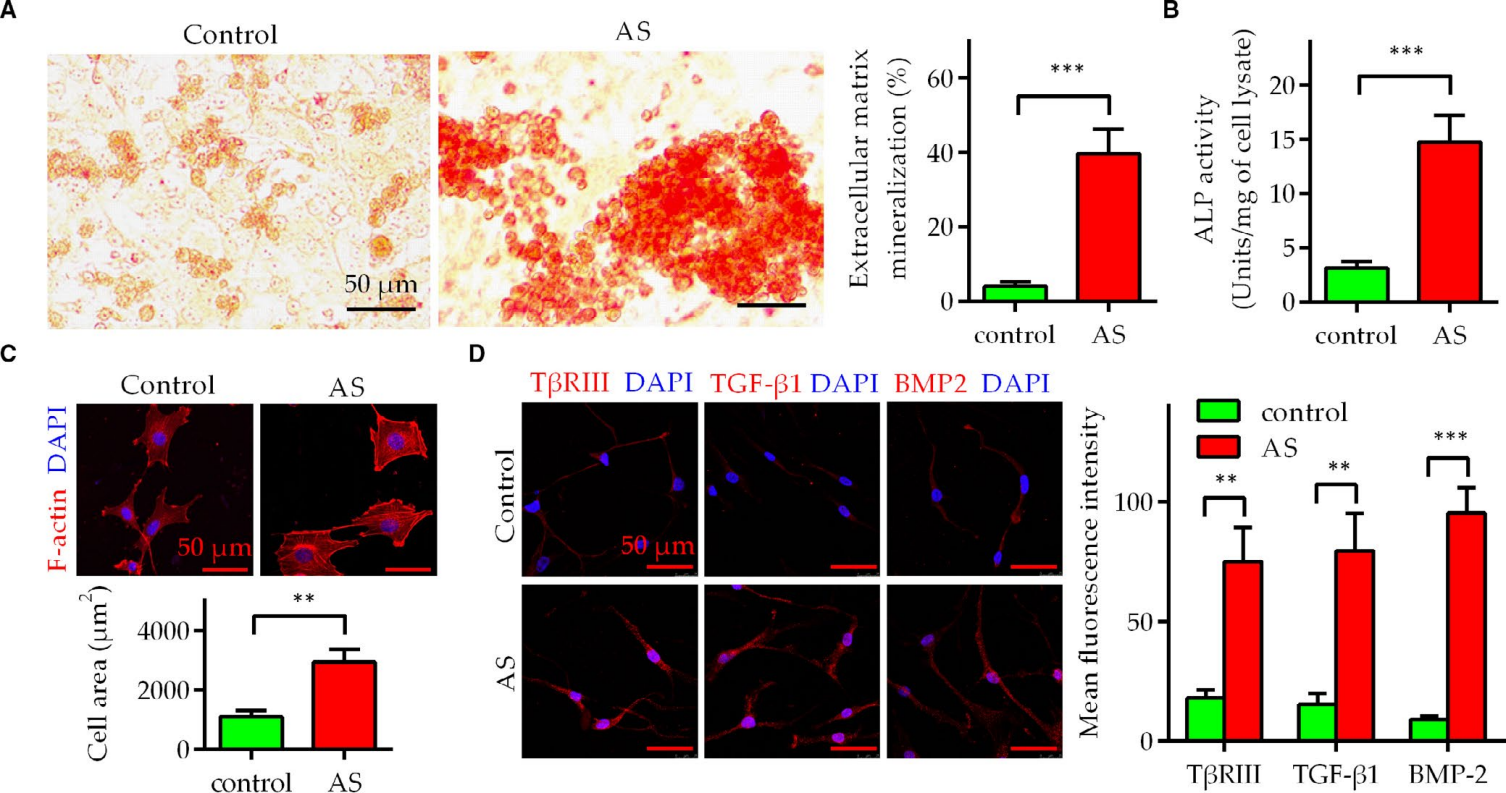
AS‐SLFs exhibit auto‐osteogenic capacity and up‐regulation of TβRIII, TGF‐β1 and BMP2. A, B, C‐SLFs and AS‐SLFs were plated in 24‐well plates (1 × 10^4^ cells/well). Then, the cells were differentiated in DMEM containing FBS (10%; m/v), β‐glycerophosphate sodium (10 mmol/L), dexamethasone (0.1 μmol/L) and vitamin C (30 mmol/L) for 14 days and further used for mineralization assays (A) or ALP activity assays (B). Bar = 50 μm. C, C‐SLFs and AS‐SLFs were seeded on glass slides in a 24‐well plate (1 × 10^4^ cells/well) for 48 h, stained with rhodamine‐phalloidin and observed via laser confocal microscopy. The cell area was measured in ImageJ (n = 50; bar = 50 μm). D, Cells cultured as described in (C) were stained for TβRIII, TGF‐β1 and BMP2 (Alexa Fluor 555; red). The mean fluorescence intensity was measured in ImageJ (n = 50; bar = 50 μm). **P < .01; ***P < .001

Fibroblasts play crucial roles in extracellular matrix (ECM) alteration, organ remodelling and tissue repair. To further investigate the role of fibroblasts in AS, we isolated and obtained C‐SLFs and AS‐SLFs. By measuring the mineralization capacity and ALP activity of both C‐SLFs and AS‐SLFs, we found that compared to C‐SLFs, AS‐SLFs showed strong auto‐osteogenic capacity (Figure 2A,B). Moreover, AS‐SLFs had a larger cell area than C‐SLFs (Figure 2C). Furthermore, the expression of TβRIII, TGF‐β1 and BMP2 was markedly up‐regulated in AS‐SLFs (Figure 2D). These results strongly suggest that up‐regulation of TβRIII, TGF‐β1 and BMP2 may mediate osteogenic differentiation of AS‐SLFs; however, more evidence is needed to confirm this hypothesis.

### 3.3 TβRIII up‐regulation facilitates the osteogenesis of C‐SLFs

**FIGURE 3:**
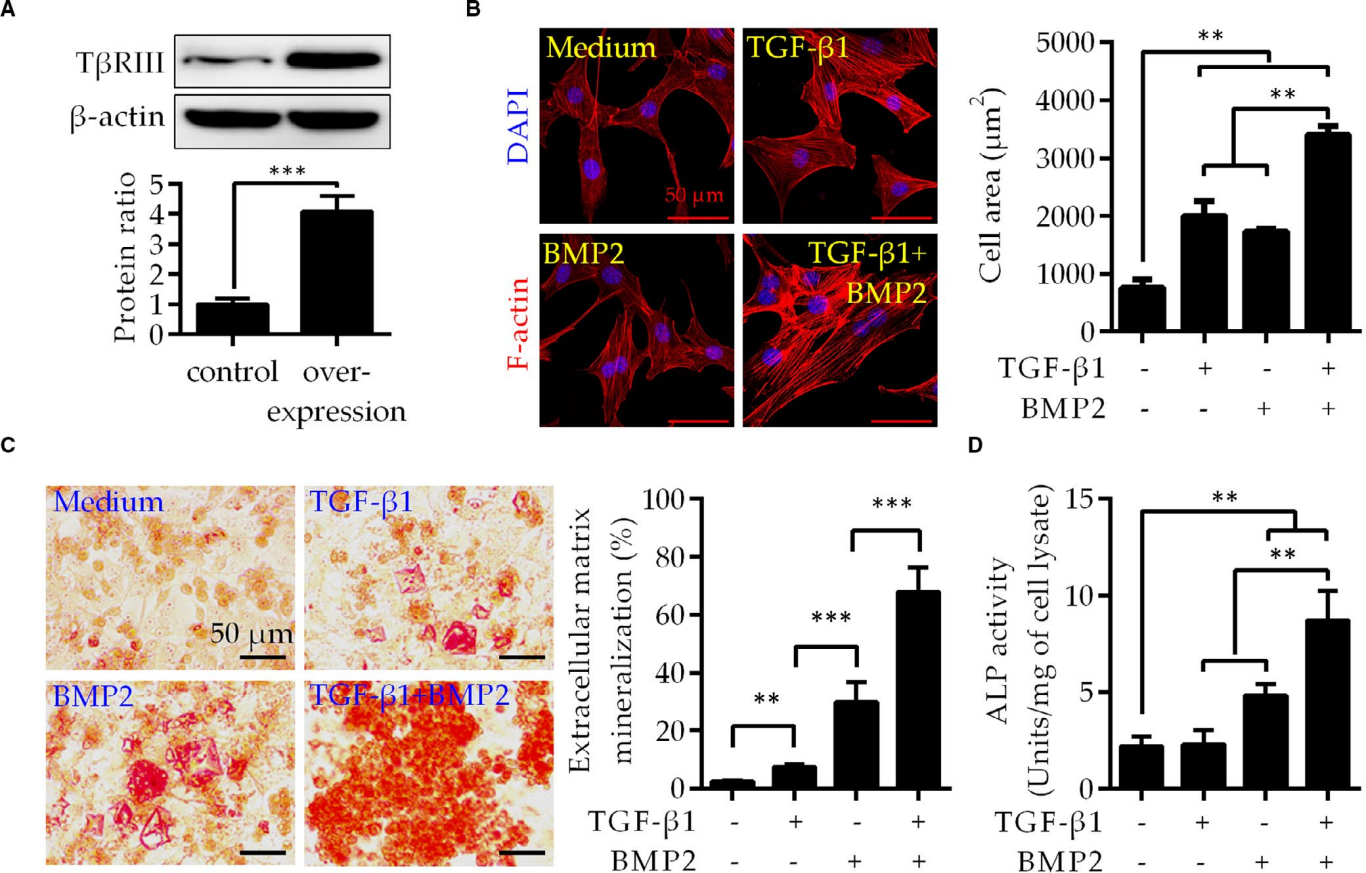
TβRIII up‐regulation facilitates osteogenic differentiation of C‐SLFs. A, C‐SLFs were plated in 6‐well plates (1 × 10^5^ cells/well) and transfected with a 3′‐Flag tagged TβRIII construct using a liposome method. TβRIII expression was assessed by IB. B, C‐SLFs with TβRIII up‐regulation were cultured as shown in Figure 2C and treated with TGF‐β1 (10 μmol/L) and/or BMP2 (50 μg/mL) for 24 h. Cells were then stained, observed and analysed as shown in Figure 2C (n = 50; bar = 20 μm). C, D, C‐SLFs with TβRIII up‐regulation were plated in 24‐well plates (1 × 10^4^ cells/well), treated as shown in (B), differentiated as shown in Figure 2A, and then further used for mineralization assays (C) or ALP activity assays (D). Bar = 50 μm. **P < .01; ***P < .001

To further confirm that up‐regulation of TβRIII, TGF‐β1 and BMP2 may induce the osteogenesis of SLFs, we constructed a C‐SLF cell line with TβRIII up‐regulation (Figure 3A). The cytoskeleton of C‐SLFs with TβRIII up‐regulation was stained with rhodamine‐phalloidin, and the effect of TGF‐β1 and BMP2 on the cell area was determined. The cell morphology of the medium group was spindle or polygonal, consistent with that of normal C‐SLFs, as shown in Figure 2C. Unsurprisingly, culture with either TGF‐β1 or BMP2 alone markedly induced cell hypertrophy compared with that in the medium group. Notably, the cell size in the group cultured with TGF‐β1 combined with BMP2 was further increased compared with that in the first three groups mentioned above (Figure 3B). Furthermore, TGF‐β1 combined with BMP2 also resulted in more mineralization nodules than medium, TGF‐β1 or BMP2 alone (Figure 3C). Consistent with these results, the combination group exhibited a synergistic effect on increasing ALP activity compared with that in the medium and single treatment groups (Figure 3D). These results indicate that the combination of TGF‐β1 and BMP2 induces the hypertrophy and osteogenic differentiation of C‐SLFs with TβRIII up‐regulation and further enhances the osteogenic capacity of AS‐SLFs.

### 3.4 TβRIII knockdown inhibits the osteogenesis of C‐SLFs

**FIGURE 4:**
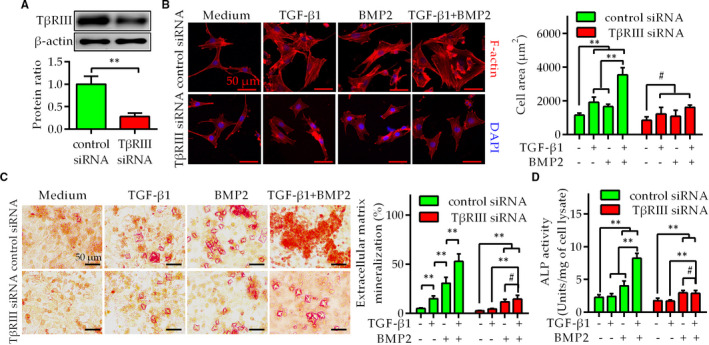
TβRIII siRNA decreases osteogenic differentiation of C‐SLFs with TβRIII up‐regulation. A, C‐SLFs with TβRIII up‐regulation were plated in 6‐well plates (1 × 10^5^ cells/well) and transfected with TβRIII siRNA or control siRNA using a liposome method. TβRIII expression was assessed by IB. B, C‐SLFs with TβRIII knockdown were treated and assessed as shown in Figure 3B (n = 50). Bar = 20 μm. C, D, C‐SLFs with TβRIII knockdown were treated as shown in Figure 3B and further used for mineralization assays (C) or ALP activity assays (D). Bar = 50 μm. **P < .01; ^#^ *P* > .05

The above data suggest that TGF‐β1 combined with BMP2 can induce osteogenic differentiation of C‐SLFs with TβRIII up‐regulation. To further confirm these findings, we constructed C‐SLFs with TβRIII knockdown using specific siRNA targeting TβRIII (Figure 4A). Consistent with the results shown in Figure 3B, TGF‐β1 combined with BMP2 showed a synergistic effect on increasing the size of C‐SLFs with TβRIII up‐regulation (transfected with control siRNA). However, neither TGF‐β1 nor BMP2 alone or in combination could change the size of C‐SLFs with TβRIII knockdown (transfected with TβRIII siRNA; Figure 4B). Consistent with this pattern, the combination treatment showed no synergistic effect on inducing mineralization nodule formation or ALP activity in C‐SLFs with TβRIII knockdown (Figure 4C,D). Collectively, these results indicate that TβRIII knockdown inhibits the osteogenic differentiation of C‐SLFs induced by TGF‐β1 plus with BMP2.

### 3.5 TβRIII up‐regulation facilitates osteogenesis via TGF‐β1‐Smad2/3 and BMP2‐Smad1‐RUNX2 signalling

**FIGURE 5:**
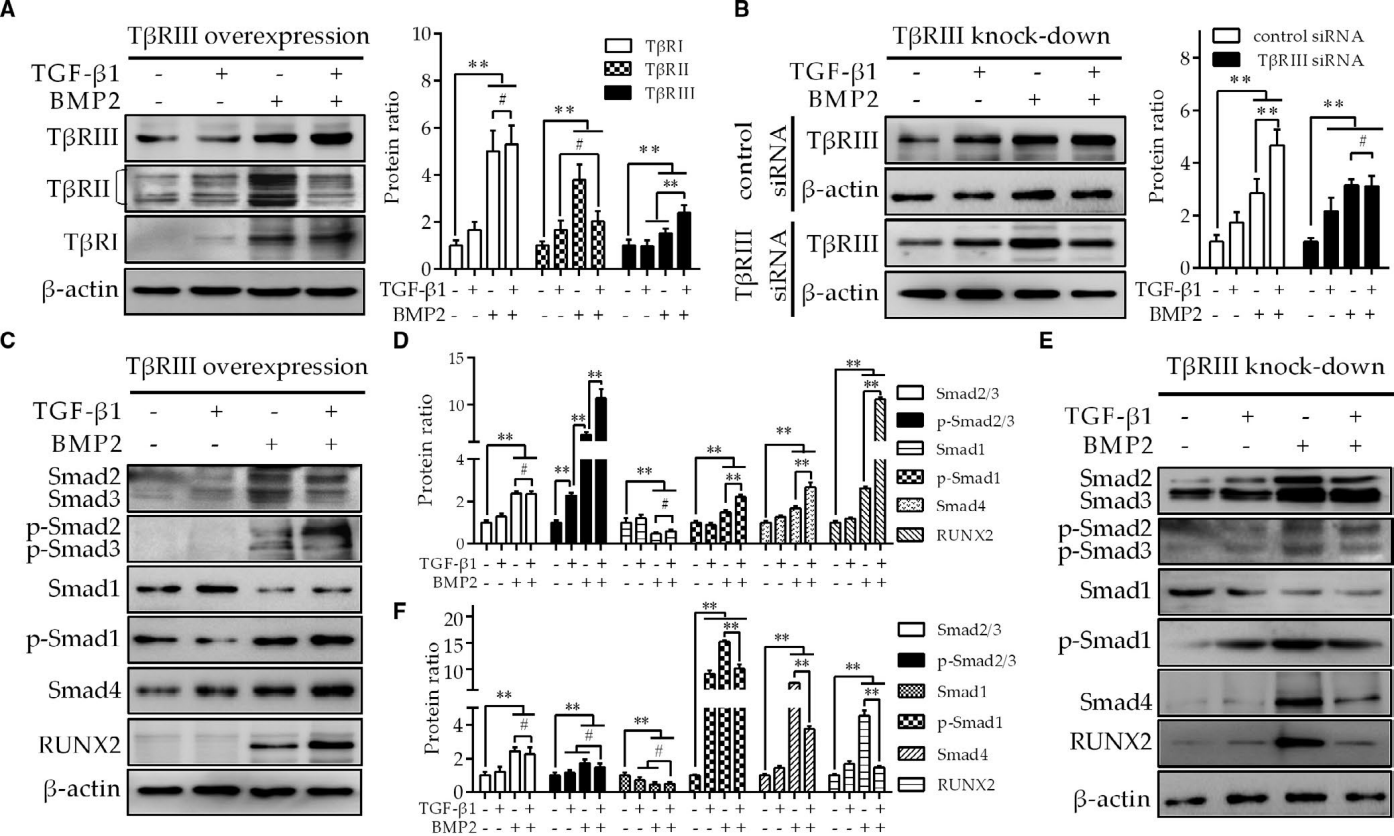
TβRIII up‐regulation facilitates the osteogenesis of C‐SLFs via TGF‐β1‐Smad2/3 and BMP2‐Smad1‐RUNX2 signalling. A, C, D, C‐SLFs with TβRIII up‐regulation were plated in 6‐well plates (1 × 10^5^ cells/well) and treated with TGF‐β1 (10 μmol/L) and/or BMP2 (50 μg/mL) for 24 h. The total lysate was extracted and used for IB with the indicated antibodies. B, E, F, C‐SLFs with TβRIII knockdown were cultured, treated and assessed as shown in (A). The histograms indicate the protein expression ratios (n = 3). **P < .01; ^#^ *P* > .05

The above results indicate that TβRIII up‐regulation is critical for the osteogenic differentiation of C‐SLFs, but the detailed mechanism remains unclear. Using C‐SLFs with TβRIII up‐regulation, we showed that compared to TGF‐β1 or BMP2 alone, TGF‐β1 in combination with BMP2 up‐regulated the expression of TβRIII but not TβRI and TβRII (Figure 5A). However, these effects were lost in C‐SLFs with TβRIII knockdown (Figure 5B).

In addition, we further observed the effect of TGF‐β1 in combination with BMP2 on Smad signalling downstream of TGF‐β1 and BMP2 in these two cell lines. TGF‐β1 in combination with BMP2 markedly increased the phosphorylation of Smad2/3 (TGF‐β1 signalling), Smad1 (BMP2 signalling) and Smad4 (the co‐Smad of both TGF‐β1 and BMP2 signalling) as well as the expression of RUNX2 (BMP2 signalling) (Figure 5C,D). However, the synergistic effects were lost in C‐SLFs with TβRIII knockdown (Figure 5E,F). These results suggest that TGF‐β1 combined with BMP2 further up‐regulates TβRIII expression in SLFs with TβRIII up‐regulation and then sequentially activates both TGF‐β1‐Smad2/3 and BMP2‐Smad1‐RUNX2 signalling, leading to cell osteogenic differentiation.

### 3.6 TGF‐β1 presents BMP2 to TβRIII, thereby increasing BMP2‐BMPR1A binding

The above data indicate that TGF‐β1 combined with BMP2 up‐regulates TβRIII‐mediated TGF‐β1 and BMP2 signalling. In addition to the expression levels, whether and how TβRIII up‐regulation contributes to the binding of TGF‐β1 or BMP2 to their own receptors are unclear. Therefore, colocalization and co‐IP assays were used to evaluate whether TβRIII up‐regulation affects TGF‐β1 and BMP2 ligand‐receptor interactions.

**FIGURE 6:**
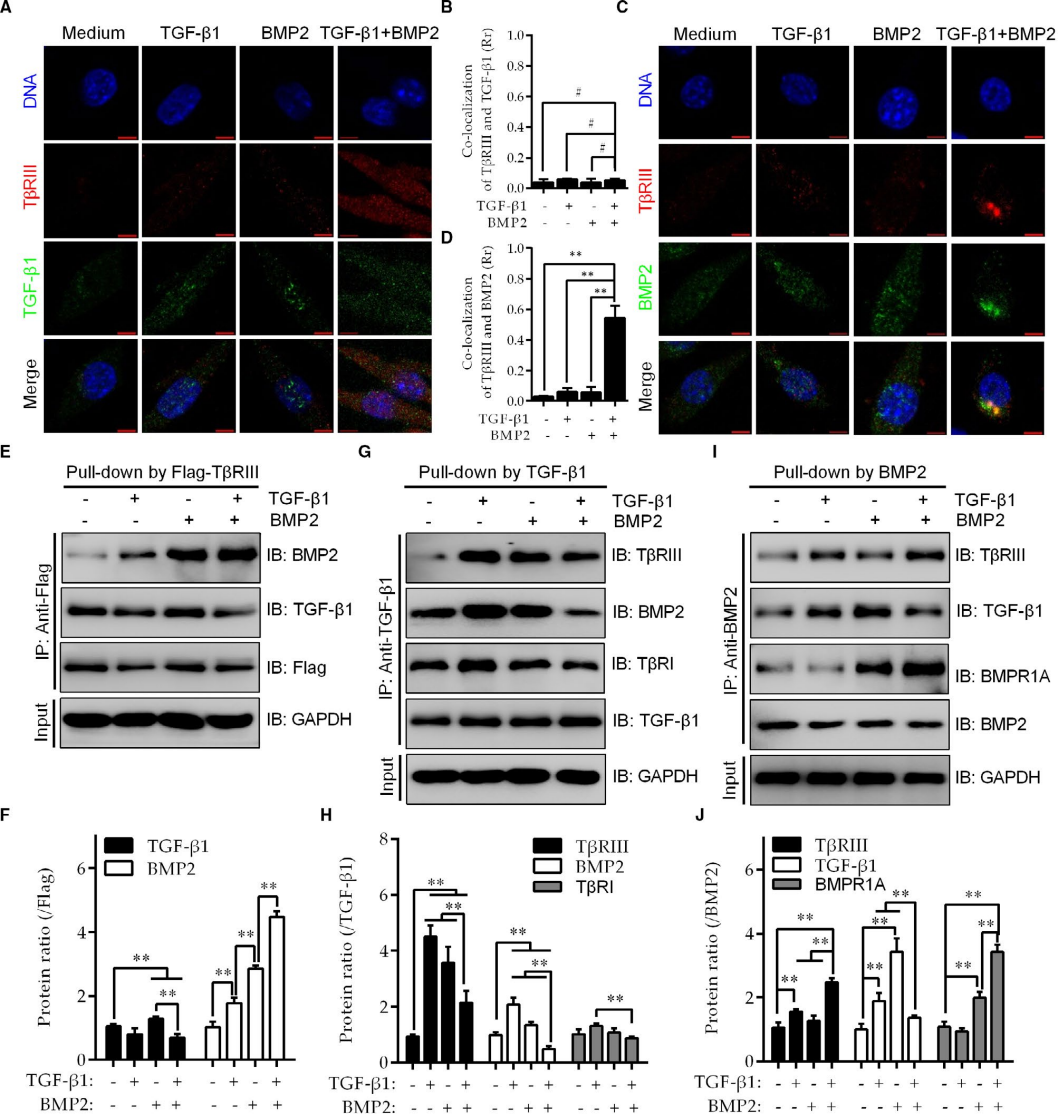
TGF‐β1 presents BMP2 to TβRIII, thereby increasing BMP2‐BMPR1A binding. A, B, C‐SLFs with TβRIII up‐regulation were seeded on glass slides in a 24‐well plate (1 × 10^4^ cells/well) and treated with TGF‐β1 (10 μmol/L) and/or BMP2 (50 μg/mL) for 24 h. Cells were then stained for TGF‐β1 (Alexa Fluor 488; green) and TβRIII (Alexa Fluor 555; red) and observed under a laser confocal microscope (bar = 5 μm). Colocalization of TGF‐β1 with TβRIII was assessed using the Pearson correlation coefficient (*Rr*) via ImageJ (n = 50). C, D, Experiments were performed as described in (A), and cells were stained for BMP2 (Alexa Fluor 488; green) and TβRIII (Alexa Fluor 555; red) (bar = 5 μm). E‐J, C‐SLFs with TβRIII up‐regulation were seeded in 10‐cm dishes (1 × 10^6^ cells/dish) and treated as in (A). The total lysate was used for immunoprecipitation (IP) using antibodies against (E, F) Flag, (G, H) TGF‐β1 or (I, J) BMP2, and the associated proteins, as indicated, were detected via immunoblotting (IB). Histograms represent the protein expression ratios (n = 3). ^#^ *P* > .05; **P < .01

In the colocalization experiments, TGF‐β1 or BMP2 was labelled green, TβRIII was labelled red, and colocalization of TβRIII‐TGF‐β1 and TβRIII‐BMP2 was observed as yellow fluorescence. Neither TGF‐β1 nor BMP2 alone or in combination increased TβRIII‐TGF‐β1 colocalization (Figure 6A,B). In addition, neither TGF‐β1 nor BMP2 alone or in combination increased TβRIII‐BMP2 colocalization; however, TGF‐β1 combined with BMP2 markedly increased TGFβR3‐BMP2 colocalization (Figure 6C,D). These results suggest that TGF‐β1 promotes the binding of BMP2 to TβRIII, but BMP2 does not affect the TβRIII‐TGF‐β1 interaction.

To further confirm the colocalization assay results, antibodies against TGF‐β1, BMP2 and Flag‐TβRIII were separately used to pull down the associated proteins in total cell lysate extracted from C‐SLFs with TβRIII up‐regulation. The results of IB on the Flag‐TβRIII immunoprecipitate showed that BMP2 combined with TGF‐β1 decreased the TβRIII‐TGF‐β1 interaction while significantly increasing the TβRIII‐BMP2 interaction, indicating that TGF‐β1 presents BMP2 to TβRIII (Figure 6E,F). Unsurprisingly, in the TGF‐β1 immunoprecipitate, BMP2 combined with TGF‐β1 did not increase the binding of TGF‐β1 to TβRIII or TβRI (Figure 6G,H). Remarkably, in the BMP2 immunoprecipitate, BMP2 combined with TGF‐β1 increased both the TβRIII‐BMP2 and BMPR1A‐BMP2 interactions, suggesting that TGF‐β1 presents BMP2 to TβRIII, thereby facilitating the recognition of BMP2 by its own receptor, BMPR1A (Figure 6I,J). Not surprisingly, no TGF‐β1, BMP2 or TβRIII band was detected in pull‐down products of anti‐mouse IgG used as a negative control (data not shown). These results suggest that TβRIII binds TGF‐β1 and BMP2 simultaneously and further indicate that TGF‐β1 presents BMP2 to TβRIII and facilitates the recognition of BMP2 by BMPR1A.

### 3.7 p‐Smad2/3, Smad4, p‐Smad1 and RUNX2 levels are increased in AS‐SLs

**FIGURE 7:**
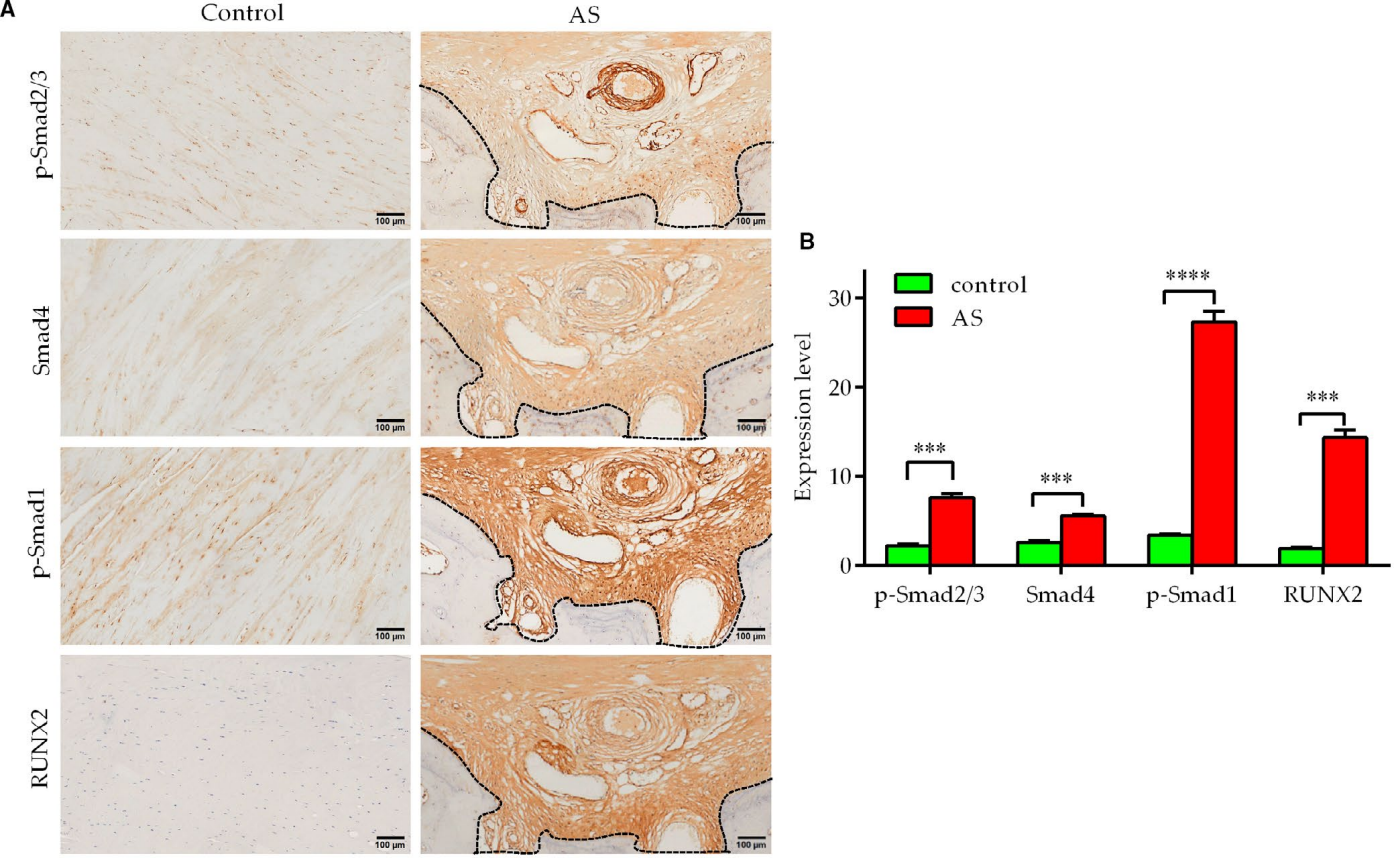
AS‐SLs from patients exhibit up‐regulation of p‐Smad2/3, Smad4, p‐Smad1 and RUNX2. A, The sections from the same specimens as described in Figure 1A were subjected to immunohistochemistry (IHC) using antibodies against phospho‐Smad2Ser465/467/3Ser423/425 (p‐Smad2/3), Smad4, phospho‐Smad1Ser206 (p‐Smad1) and RUNX2. The sections were examined under a light microscope (bar = 100 μm). The dotted lines indicate the edge of the ectopic bone. B, Expression levels were detected by measuring positive staining area in ImageJ and were calculated by normalization to the control. ***P < .001 and ****P < .0001 (n = 3)

Paraffin sections of C‐SLs and AS‐SLs (the same sections as shown in Figure 1) were used for IHC to assess the activation of TGF‐β1‐Smad2/3 and BMP2‐Smad1‐RUNX2 signalling. No large areas of brown coloration were observed in C‐SLs, suggesting that the levels of p‐Smad2/3, p‐Smad1, Smad4 and RUNX2 were decreased. However, the immunohistochemical results showed that the levels of p‐Smad2/3, p‐Smad1, Smad4 and RUNX2 were increased in the tissue near the ectopic bone in the contiguous sections (Figure 7). The results further indicate that TGF‐β1‐Smad2/3 and BMP2‐Smad1‐RUNX2 signalling are activated in AS‐SLs.

## 4 DISCUSSION

Numerous reports indicate that inflammation, bone erosion and cartilage formation are the basic characteristics of AS pathology. Ectopic osteogenesis within the SL is the key risk of AS and is the main cause of joint fusion/stiffness and mobility loss. As shown in Figure 1, significant fibre breakage and disorganization, with collagen deposition, angiogenesis and ectopic bone formation, were observed in AS ligaments. The data presented here suggest that AS‐SLFs have a larger cell area and exhibit obvious self‐osteogenic capacity in vitro, consistent with other reports.^12^ The understanding of the molecular mechanisms underlying new bone formation in AS has significantly improved but remains incomplete. Here, we demonstrate that TGF‐β1 combined with BMP2 can induce the osteogenesis of AS‐SLFs by acting on up‐regulated TβRIII, thus resulting in excessive activation of both TGF‐β1/Smad and BMP2/BMPR1A/Smad/RUNX2 signalling. These findings establish a novel pathway downstream of TβRIII in AS progression that contributes to bone formation by regulating the function of TGF‐β1 and BMP2.

The key role of inflammation in regulating bone remodelling has been extensively reported, and spatiotemporally controlled release of inflammatory signals and factors is essential for regulating bone remodelling.^13^ Dysregulated inflammation leads to increased bone resorption and suppressed bone formation in bone diseases such as arthritis and fracture, but its detailed mechanism in promoting ectopic osteogenesis in AS is unclear. Typically, proinflammatory cytokines, including tumour necrosis factor‐α (TNF‐α), IL‐1, IL‐11, IL‐6 and IL‐17, promote bone resorption by enhancing osteoclast differentiation and activity and/or inhibiting osteoblast differentiation, collagen synthesis and bone formation, while anti‐inflammatory cytokines, such as TGF‐β, IL‐10 and IL‐13, show the opposite effects. Recent evidence suggests that a series of inflammatory mediators, such as TGF‐β, interleukin and TNF‐α, not only play a key role in AS but also affect osteoclast and osteoblast activity and lead to bone remodelling. Therefore, some clinical studies have shown that inflammation inhibitors, such as monoclonal antibodies against TNF‐α and new biological inhibitors targeting IL‐17/IL‐23, could slow radiological progression in AS patients.^14^ However, their impact on slowing bone structural damage is still not clearly established.^15^, ^16^ Inflammatory cytokines such as TNF‐α and IL‐1β have also been reported to play a coordinated role in regulating osteoblast differentiation in vitro and in vivo.^17^ In addition to the proinflammatory factors TNF‐α and IL‐6, the following factors play a significant role in osteoblast differentiation: Wnt/wingless protein family members, BMP, TGF‐β, fibroblast growth factor (FGF) and insulin‐like growth factor (IGF).

The TGF‐β superfamily comprises a group of polypeptide factors with similar structures, including TGF‐β, activin/inhibitors, BMPs and growth differentiation factor (GDF), that play key roles in inflammation, bone reconstruction and cancer progression.^18^ Both TGF‐β1 and BMP2 belong to the TGF‐β superfamily and are involved in inflammation and bone formation. They are important osteogenic mediators and contribute to the progression of multiple diseases,^19^ including AS.^2^, ^3^, ^20^ Previous studies indicate that TGF‐β and BMP2 have synergistic effects on promoting osteogenic differentiation,^21^, ^22^, ^23^ but the detailed mechanism remains unclear. Wang and He noted that TGF‐β3 and BMP2 have a synergistic effect on regulating the osteogenic differentiation of bone marrow mesenchymal stem cells but did not propose a specific mechanism.^23^, ^24^ As our results in Figure 2 show, TGF‐β1 combined with BMP2 further induces the hypertrophy and osteogenic differentiation of C‐SLFs and may participate in the osteogenic differentiation of AS‐SLFs. Furthermore, our study further unveils the mechanism by which TGF‐β1 synergizes with BMP‐2 to promote osteogenic differentiation in AS.

TβRIII (also known as β‐proteoglycan) was originally thought to be an auxiliary receptor for members of the TGF‐β superfamily, including TGF‐β and BMP2, and to enhance their cellular effects.^6^, ^7^ In endocardial cells, TβRIII is required to activate the Par6/Smurf1/RhoA pathway via TGF‐β and BMP‐2 to induce EMT and invasion.^7^, ^25^, ^26^ TβRIII binds all three TGFβ ligands and BMP2, but its contribution to AS is unknown. Our research further indicated that the expression of TGF‐β1, BMP2 and TβRIII was significantly increased in AS‐SLFs compared with C‐SLFs. As shown in Figure 5A, TGF‐β1 combined with BMP2 significantly up‐regulated the expression of TβRIII but not TβRI or TβRII. Subsequent findings indicated that after up‐regulation of TβRIII expression in C‐SLFs, TGF‐β1 combined with BMP2 further induced cell hypertrophy and mineralization nodule formation compared with these effects in the single treatment groups. However, the synergistic effects were lost in C‐SLFs with TβRIII knockdown, further suggesting that TβRIII up‐regulation may participate in the osteogenic differentiation of AS‐SLFs, which has rarely been reported.^7^, ^19^

Additionally, our experiments further confirmed that TGF‐β1 promotes the binding of BMP2 to TβRIII, but BMP2 does not affect the TβRIII‐TGF‐β1 interaction. Moreover, in the presence of TGF‐β1, up‐regulated TβRIII presents BMP2 for binding to TβRIII, which facilitates the recognition of BMP2 by BMPR1A and then may be have a role in enhances the osteogenesis of AS‐SLs. These data further demonstrated another possible important mechanism underlying the TβRIII‐mediated function of TGF‐β1 and BMP2 that contributes to osteogenic differentiation in AS that has not been reported.

Multiple signalling pathways, such as the BMP‐Smads, Wnt/β‐catenin, Notch, Hedgehog and FGF signalling pathways, have been indicated to play critical roles in the regulation of osteogenic differentiation. These signalling pathways not only have complex regulatory mechanisms but are also interrelated and influence each other, thus forming a more complex and sophisticated regulatory network to regulate osteogenic differentiation. To our knowledge, both TGF‐β1 and BMP‐2 activate Smad signalling, and phosphorylated Smads translocate into the nucleus to promote the expression of genes involved in osteoblast differentiation.^27^, ^28^ Typically, BMP‐2 primarily utilizes Smad1, whereas TGF‐β1 utilizes Smad2/3.^27^, ^29^ Herein, we demonstrate that TGF‐β1 combined with BMP2 up‐regulates TβRIII expression in SLFs and sequentially activates both TGF‐β1‐Smad2/3 and BMP2‐Smad1‐RUNX2 signalling, leading to cell osteogenic differentiation. In addition, the immunohistochemical results indicated TGF‐β1‐Smad2/3 and BMP2‐Smad1‐RUNX2 signalling activation in paraffin sections of human AS‐SLs but not C‐SLs. However, further animal experiments are needed.

## 5 CONCLUSIONS

In conclusion, we demonstrated that TGF‐β1 combined with BMP2 may participate in the osteogenic differentiation of AS‐SLFs by acting on up‐regulated TβRIII, thus resulting in excessive activation of both TGF‐β1/Smad and BMP2/BMPR1A/Smad/RUNX2 signalling. These data force us to reconsider the interactive roles and contributions of these ligands in the activation of canonical signalling pathways through TβRIII in AS.

## CONFLICT OF INTEREST

The authors declare no conflict of interest.

## AUTHOR CONTRIBUTION

**Ying Zhang:** Conceptualization (lead); Data curation (lead); Formal analysis (lead); Funding acquisition (lead); Project administration (lead); Validation (equal); Writing‐original draft (lead); Writing‐review & editing (lead). **Wu‐gui Chen:** Data curation (equal); Formal analysis (equal); Investigation (equal); Methodology (lead); Software (lead); Writing‐original draft (equal); Writing‐review & editing (equal). **Si‐zhen Yang:** Data curation (equal); Formal analysis (equal); Investigation (equal); Writing‐original draft (equal). **Hao Qiu:** Formal analysis (supporting); Software (supporting); Supervision (supporting); Writing‐review & editing (equal). **Xu Hu:** Data curation (supporting); Formal analysis (supporting); Writing‐review & editing (supporting). **Yi‐yun Qiu:** Data curation (supporting); Formal analysis (supporting); Writing‐review & editing (supporting). **Xuan Wen:** Data curation (supporting); Formal analysis (supporting); Writing‐review & editing (supporting). **Yue Zhou:** Project administration (equal); Resources (supporting); Writing‐review & editing (supporting). **Tong‐wei Chu:** Conceptualization (lead); Project administration (lead); Resources (equal); Writing‐original draft (equal); Writing‐review & editing (equal).

## ETHICAL APPROVAL AND CONSENT TO PARTICIPATE

This study was approved by the Research Ethics Committee of Xinqiao Hospital, and informed consent was obtained from all participants.

## CONSENT FOR PUBLICATION

Written informed consent for publication was obtained from all participants.

## Data Availability

The datasets used and/or analysed during the current study are available from the corresponding author on reasonable request.
